# Cholera epidemiology analysis through the experience of the 1973 Naples epidemic

**DOI:** 10.1515/med-2024-1029

**Published:** 2025-09-04

**Authors:** Bifulco Maurizio, Di Zazzo Erika, Pagano Cristina, Martini Mariano, Orsini Davide

**Affiliations:** Department of Molecular Medicine and Medical Biotechnology, University of Naples “Federico II”, Naples, Italy; Department of Medicine and Health Sciences “V. Tiberio”, University of Molise, Campobasso, Italy; Department of Health Sciences, University of Genoa, Genoa, Italy; University Museum System of Siena (SIMUS), History of Medicine, University of Siena, Siena, Italy; Cardarelli Hospital, Campobasso, Italy

**Keywords:** history of cholera, environmental ethics, global health, vaccination, anthropization

## Abstract

**Background and objective:**

The World Health Organization (WHO) appeal of January 15, 2024, stated “The current number, size and concurrence of multiple outbreaks, the spread to areas free of cholera for decades and alarmingly high mortality rates present a major threat to global health security.” The current state is extremely worrying, considering the difficulties of countries in dealing with cholera epidemics due to the lack of funding and the difficulty in oral cholera vaccine production and administration. This study aims to analyse the past and current influence of anthropization on cholera onset.

**Methods:**

We analysed the literature, particularly of the last 5 years, on the influence of human actions that impact the spread of cholera.

**Results:**

The epidemiological data published by WHO and the available literature highlight a strong impact of human actions on the epidemic spread of cholera, the government’s difficulty in making decisions on epidemic prevention or containment, and the fear of the population.

**Conclusions:**

Cholera should be considered an anthropogenic disaster, considering the historical health analysis of the cholera epidemics in Italy in the last two centuries and in southern Italy and in Naples in 1973.

## 1 Overview of the current situation of cholera epidemics

The year 2024 opened with an alarming appeal from the World Health Organization (WHO) regarding cholera: “The current number, size and concurrence of multiple outbreaks that have spread to areas free of Cholera and the alarmingly high mortality rates present a major threat to global health security” [1].

A wave of cholera epidemics without precedent occurred in the 2-year period of 2022–2023, even in countries that have not reported cholera for many years [2].

The data, not definitive, relating to 2023 and updated to December 15th, recorded 667,000 cholera cases and over 4,000 deaths. These numbers are extremely worrying, especially when compared to those of past years, which in turn are increasing compared to previous periods [3]. In 2022, the global number of cholera cases reported to WHO was 472,697, compared to 223,370 cases in 2021 [4].

The WHO African Region remains the most affected region worldwide, with 17 countries reporting cholera cases throughout 2023.

The current ability to deal with cholera epidemics is severely limited by the lack of funding at a global level due to the enormous expenses faced by the COVID-19 pandemic. In addition, there are several difficulties related to the production and distribution of oral vaccines against cholera.

This places a huge burden on vaccine manufacturing as the large requests means that the supplies dedicated to preventive campaigns are limited. In 2013, 12 countries submitted requests for significant supplies of anti-cholera vaccines because they were affected by very virulent cholera epidemics. “A total of 33 million doses were dispatched, fully allocating the available stockpile. […] In December alone, four new requests, totalling 13.6 million doses, were submitted to the International Coordinating Group (ICG) from Ethiopia, Sudan, Zambia, and Zimbabwe. […] As of 9 January 2024, the global OCV stockpile stands at 1.1 million doses, which are available for allocation but not yet committed” [5].

## 2 Cholera epidemics between the nineteenth and twentieth centuries: Fear and lack of knowledge of the causes

Endemic in Asia, and mainly in the delta of the Ganges (India), it remained narrowed to that geographical area until the beginning of the 1800s. It first emerged from the Sundarbans Forest of the Bay of Bengal, in the Ganges delta, where the bacterium *Vibrio cholerae* had probably been mutating for millennia. The microorganism is found naturally in the environment in some coastal areas, wastewater, and brackish waters, where shellfish often carry the infection. It subsequently reached the Mediterranean and Europe as new trade routes were opened between the East and West [2].

Cholera is an acute enteric infection caused by the bacterium *V. cholerae*, belonging to the genus *Vibrio*, of the family Vibrionaceae. The genus *Vibrio* includes pathogens to humans (*V. cholerae*, *Vibrio parahaemolyticus*, *Vibrio vulnificus*), opportunistic pathogenic species (*Vibrio alginolyticus*, *Vibrio mimicus*, etc.) and other saprophytic isolates from the sweet waters of the sea (*Vibrio anguillarum*, *Vibrio fischeri*, etc.), which can be confused with human vibrios [6].

The *V. cholerae* was observed for the first time under microscope in 1854 by the Italian anatomist Filippo Pacini (1812–1883) in Florence. In 1884, it was isolated in Egypt and studied in detail by the German physician Robert Koch (1843–1910) [7].

During the nineteenth and twentieth centuries, the disease spread globally beyond Asia seven times, referred to as the cholera pandemics. The first cholera pandemic started in August 1817 when the British government received a report of a “malignant disorder” in the Sundarbans, killing 20–30 people a day, and during the following weeks, 10,000 people died. Thereafter, the disease spread across the countries reaching eastwards and westwards of Nepal, Iran, Iraq, Afghanistan, Oman, Thailand, Burma, China, and Japan. The incidence of cholera decreased, but a second one began in 1826, and the source was again the Ganges Delta, and the disease spread fast again, but also moving towards the United States, Europe, and Egypt. Cairo and Alexandria recorded 33,000 deaths a day.

Subsequently, new pandemics began in 1829, 1831 (when it devastated the great trading city of Astrakhan), 1852, 1863, 1881, 1889, and 1961 [8], the last persisting until now [9,10].

Cholera appeared in Europe in the first half of the ninetenth century, because of the opening of new communication routes between the East and West: its journey began in India in 1817, in a very difficult period for the Indian subcontinent, which was hit by floods and continuous famines. The large masses of people who, on this occasion, began to move from the areas affected by famine caused the disruption of an environmental balance, which determined the spread of the cholera bacterium outside its natural habitat. This difficult situation was certainly exacerbated by the appearance of a more virulent variant of the disease, which inexorably affected that large mass of fugitives who hoped to leave hunger behind and, instead, increasingly malnourished and in precarious hygienic-sanitary conditions, ended up a victim of the first cholera pandemic (1817–1823).

However, it was the second pandemic that originated in the 1820s that fully affected Europe, causing massacres among the poorest and most defenseless population [11].

When cholera first appeared in Europe, at the end of the 1820s, the advanced world began with great concern to acknowledge. Physicians in France, Britain, and Russia began studying the disease as a matter of urgency. It progressively followed the trade route and appeared in new places only after the arrival of people from the infected area.

The cholera origin from distant and unknown countries, the spreading speed and the sudden death of infected people had a very strong impact on collective imagination, fueling anxiety and terror, similarly to COVID-19 pandemic [12].

In 1836, cholera reached the south of the Italian peninsula. It hit Naples, the capital of the Kingdom of the Two Sicilies, with great force, in two successive phases: between October 1836 and March 1837 and then from April 1837 to October 1837. In the first phase, it infected 10,361 people, of which 5,669 (54.7%) died. Much more serious was the second epidemic in which 21,784 people were infected and 13,810 died, with a lethality rate of 69.39% [13].

In Italy, cholera struck with great force between 1884 and 1886, arriving from France and, in particular, from the port city of Toulon, where a ship from Indochina had landed carrying soldiers suffering from an anomalous form of gastroenteritis, probably identified as cholera.

To prevent the spread of cholera in the various italian regions, it was decided to prohibit access to the ports for ships coming from Toulon and the infected areas. The first case was recorded in Piedmont; the infection then spread to Liguria, Lombardy, Tuscany, and Veneto, and in August, it reached Naples and then Calabria, invading all of Southern Italy. Naples was certainly its epicenter: the frequent and constant commercial relations with the French ports, the living conditions of a large part of the population who lived in the port area, the inadequate sewage system, the poor supply of drinking water, as well as the disorganization of the hospitals were the main causes of the devastation of Naples and the surrounding areas.

Consider that, in 1884, the conditions of the Neapolitan water network were truly terrible: the water, undrinkable and polluted, arrived from two aqueducts: the Bolla and the Carmignano (the most recent dated back to 1627), which had not received any expansion or maintenance work. In the homes, especially those in the lower city (which housed around 130,000 people in difficult hygienic conditions), there was no type of sewage system, and the only outlets were the stairs and courtyards of the homes, and everything was poured out along the roads.

To avoid the continuation of the epidemic and any new epidemics, the “gutting” of the ancient part of Naples was ordered, with the demolition of the degraded neighbourhoods and the construction of new ones [14].

It is estimated that this cholera epidemic caused in the Neapolitan area around 8,000 victims, two-thirds of the deaths that affected the whole of Italy on that occasion [15].

The disease returned to hit the regions of Southern Italy, especially in Puglia, Sicily, and Campania, between 1910 and 1911, fortunately causing fewer deaths. On both occasions, cholera spread due to the poor hygienic conditions of the affected cities. The same causes can be remembered for the epidemics of the 1910s. The government of the time commissioned the hygienist Achille Sclavo (1861–1930) to coordinate all actions to eradicate or at least limit the spread of cholera, which had hit Southern Italy, particularly in Puglia and Sicily [16,17]. During that period (on that occasion), Sclavo published the writing on the problem of sewage in Puglia with special attention to the biological purification of sewer water [18].

For reasons that are not easily identifiable, probably linked to the serious droughts that hit many African regions in the summer of 1973, unfortunately the spread of cholera was recorded in some African countries on the Mediterranean coast, especially in Tunisia and Algeria; immediately afterwards, reports of isolated imported cases with local spread began in various European countries such as England, France, Sweden, and Germany with 25 cases. Beginning on August 26, cholera also affected Italy with 277 bacteriologically verified cases (period August-October 1973), mainly in Campania (130 cases and 15 deaths) and Puglia (125 cases and 7 deaths) [19]. Cholera was again recorded with a high percentage in the city of Naples.

Exactly 30 years ago, in 1994, the first cases of cholera were recorded in the city of Bari. The latest cases reported in Italy were recorded in 2019 [20] (one case) and in 2023 (one case).

In both cases, although there were bacteria in the patients’ feces, *V. cholerae*, these were not the strains responsible for the disease.

The transmission of the bacterium and, consequently, of the disease is closely linked to the impossibility of accessing clean water, the consumption of contaminated food, and in general, poor hygienic-sanitary conditions and the poor management of sewage systems and drinking water. This is evident in all those places where, due to a lack of treatment and prevention or due to wars or earthquakes that destroy plants and infrastructures, preventing access to clean water, cholera forcefully returns to infect populations, often already suffering from hunger and other diseases.

## 3 Cholera in Naples in 1973

Fifty years have passed since the epidemics of cholera, which, between August and November 1973, hit Naples [21].

The epidemic aroused disbelief and fear, although the number of cases was limited. It was not possible to understand how such a disease could affect an industrialized country like Italy. At the same time, the memory of past epidemics of cholera caused great concern among the population.

On August 29, 1973, the Neapolitan newspaper “Il Mattino” announced the epidemic, that caused seven deaths (five in Torre del Greco and two in Naples) and hospitalization of over fifty infected people. To limit the spread, authorities banned the consumption of raw fish and shellfish, which was the likely soure of cholera.

Although the episode is roughly referred to only as the city of Naples, it was a social, political, and economic phenomenon, which had significant consequences throughout the national territory. In particular, the pandemic affected the coastal regions of Campania and Puglia and the province of Cagliari in Sardinia in 1973 [22], and dozens of cases were also recorded in Rome, Milan, Florence, and Bologna. “The epidemic became a national threat.” The emergency even extended to the peripheral area of Rome (plagued in its own way by hygienic-social problems not dissimilar to the capital of Campania), just as threats of contagion were recorded in several parts of the country [23].

Thanks to an extraordinary vaccination campaign, the epidemic was contained within a few weeks. Official data reported 277 infected and 24 deaths, mostly concentrated in Naples, due to the consequences of infection with *V. cholerae* type 1 “The Tor.” It was ascertained that the strain responsible for the epidemic was widespread in muddy marine waters, contaminated by *V. cholerae* due to the inadequacy of the sewage system (whose last modernization dated back to the years of the restoration following the tragic epidemic of cholera that occurred between 1884 and 1887), along with spills of industrial processing waste. The sewerage system was divided into an impressive number of uncovered collection channels and dozens of discharges (authorized and illegal), which were released directly into the sea, the sewage produced by a city which, in just under 20 years, had doubled its population and its territorial extension [23]. Yet, in August 1973, many people on the coast of Naples challenged the absolute ban on bathing, diving into the sea.

The Health Care System was absolutely unprepared to deal with the epidemic at the time, so much so that the reports published by “The Times” and “Le Monde” described the confusion, improvisation, and approximation that characterized the Cotugno hospital in Naples during the emergency epidemic [23]. Within a short time, fear took possession of the Neapolitan citizens and the whole of Italy due to a pressing yet approximate narrative of the Media.

Thanks to the mass vaccination campaign, which was organized in a short time and began on September 1, 1973, it was possible to contain the number of infected people and, above all, the number of victims. The strategy of spreading vaccination extensively and across the entire population was, without a doubt, one of the most effective and successful interventions, as history had already taught us [24,25,26]. They were vaccinated in pharmacies, in the sports hall, and in the political party sections. Important support also came from NATO healthcare personnel: U.S. servicemen from the Environmental Preventive Medicine Unit (EPMU) No. 7 of the Sixth Fleet, present in Naples, vaccinated the population using the pistol syringes already used for mass vaccinations during the Vietnam War [27].

**Figure 1:**
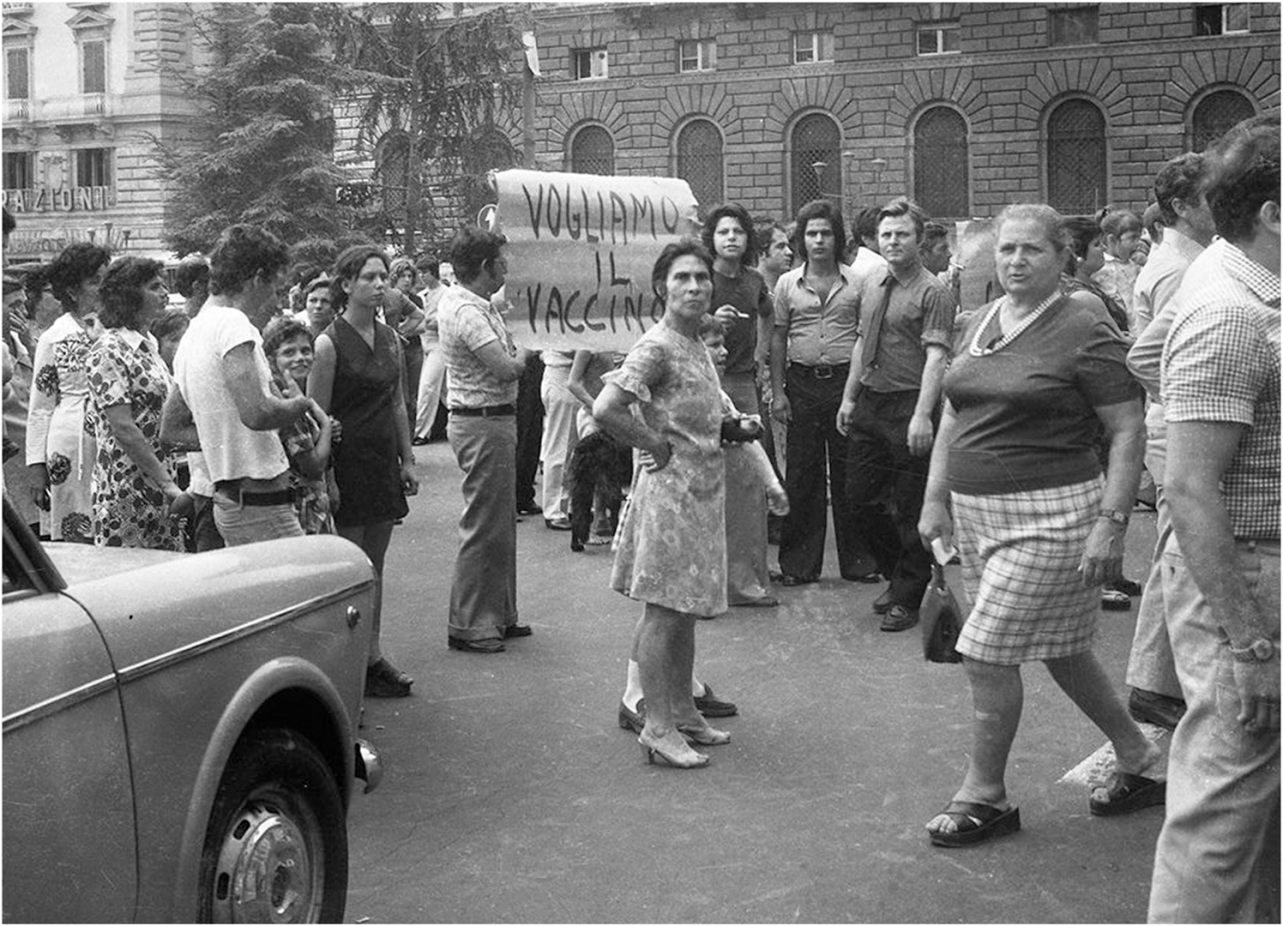
Protests in Naples in 1973 due to the lack of vaccines (public domain image).

Despite the disorganization, also denounced by protests from citizens (Figure 1), as of September 5, 1973, one million two hundred thousand people had already been vaccinated and despite the difficulties dictated by the complex contingency, the confusion created and the emergency, it was possible to set up effective and immediate communication without particular resistance in terms of uncertainty and vaccination hesitancy on the part of the entire population [28,29,30].

**Figure 2:**
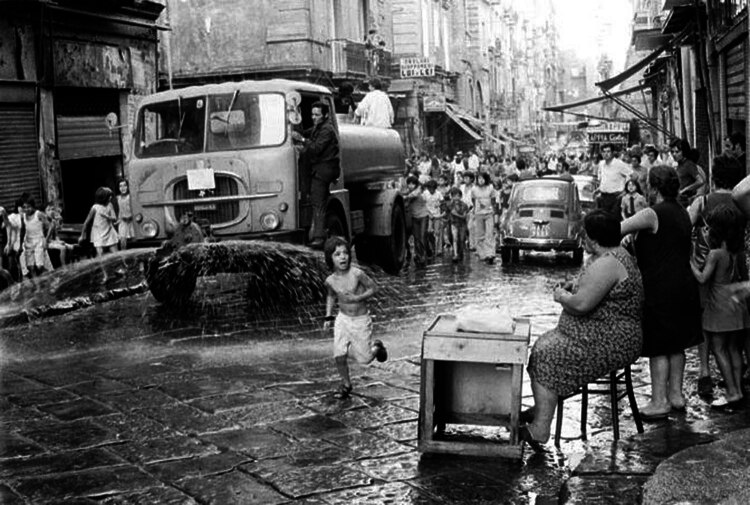
Disinfestation of the streets of Naples during the cholera epidemic of 1973 (public domain image).

The vaccination was also accompanied by specific hygiene actions, such as the extraordinary disinfection of the city’s streets with 1,500 tons of disinfectant (Figure 2), and the temporary closure of social places. In Naples, the last case was recorded on September 19, 1973, and the epidemic could be considered over on 12 October of the same year.

## 4 Similarities and differences between past and present: Research objective and methodology

Through the analysis and comparison of cholera epidemics of the nineteenth and twentieth centuries, the article aims to analyse past and current influences of human actions in favouring the onset of cholera epidemics.

Considering the recent outbreak of cholera epidemics in many countries, we performed a narrative review by analysing WHO reports and current available scientific literature of the last 5 years. Particularly, we investigated the impact of human activities, both direct as the wars ongoing in different regions of the world where cholera is dangerously reappearing, both indirect as famines, droughts, floods due to major climate changes attributable to anthropization, on cholera epidemic spreading. The search was performed in the PubMed-Medline in February 2024.

## 5 Worrying results: Cholera, a disease of iniquity, is a consequence of humanity’s wrong action

“Cholera is an ancient disease that remains a public health problem in many impoverished locations around the world. Overcrowding, poverty, insufficient water and sanitation facilities increase the risk for Cholera outbreaks” [10].

During the nineteenth-century epidemics, that of Naples in 1973, and the epidemics that have been affecting many areas of the Earth in recent years, the spread of cholera can be traced back to wrong actions carried out by man.

Therefore, one of the findings of this study is that cholera is not a natural catastrophe but must be considered in most cases an anthropic disaster. The epidemic in Naples in 1973 dramatically brought out a complex set of submerged but characterizing elements of the economic, social, and hygienic-health conditions of Naples as well as most of Southern Italy. Naples was, in those years, a metropolis in which heterogeneous social strata coexisted, marked by profound inequalities, a city in which the presence of gastrointestinal diseases, such as typhoid and viral hepatitis, was endemic in a population that lived in an urban space among the most overcrowded in the world. Similarly, many of the cholera epidemics in recent years are affecting poor populations living in complex socio-economic situations, who are often affected by famine, war or environmental disasters.

“Cholera remains a disease of inequity, disproportionately affecting the world’s poorest and most vulnerable populations” [1].

In addition, cholera epidemics are also facilitated by climate change, which, albeit indirectly, is attributable to the actions of contemporary society [31].

Considering the inability of the governments of various countries to counteract the worsening of climate changes, the increasing migration rate, and the worsening of living conditions also due to the lack of access to clean water, cholera epidemics will further spread.

Recent studies have linked the increase in sea water temperatures and cholera epidemics. Vibrios are in fact bacteria that prefer warm marine waters. Their global increase is provoked by marine warming.

“Climate change is a major driver of infectious disease dynamics through changes in temperature and precipitation. The effect of climate change on infectious diseases disproportionately affects low- and middle-income countries (LMIC)” [32,33].

Many researchers are warning of the impact that climate change sensitive to greenhouse gases (GHGs) may have on the emergence and re-emergence of infectious diseases [34]. A recent study has established that “58% (that is, 218 out of 375) of infectious diseases confronted by humanity worldwide have been at some point aggravated by climatic hazards; 16% were at times diminished. […] The human pathogenic diseases and transmission pathways aggravated by climatic hazards are too numerous for comprehensive societal adaptations, highlighting the urgent need to work at the source of the problem: reducing GHG emissions” [35].

## 6 Conclusions

Based on the results of our analysis of WHO reports and currently available scientific literature regarding cholera epidemics, it is possible to conclude that climate change, rapid urbanization, wars, and above all, the changing patterns of use of large spaces on our planet will increase the risk of disease outbreaks in the coming decades. In particular, climate change may alter the range of pathogens, allowing infections, particularly vector-borne infections, to expand into new locations. A new era is therefore opening for infectious diseases.

Through the memory of the last great epidemic that hit Italy, we believe that it is current and necessary to re-propose the theme of cholera prevention in a historical moment in which this disease is returning to affect increasingly larger areas of the Earth.

Similarly, to the cholera epidemic of Naples in 1973, the disease retains specific elements attributable to the lack of hygiene and water contamination. However, to this, there are equally, if not more dangerous, contributory causes linked to environmental problems and more generally to anthropization.

The effects of climate change, caused by the set of transformation and alteration interventions that humans are carrying out at the expense of the Earth to bend it to his own interests, also have important repercussions on human health and on public health as a whole [36].

“Climate change can alter environmental temperature, wind, and precipitation patterns, which can indirectly affect pathogen distribution, vector reproduction rates, and transmission media. These effects then further determine regional shifts in seasonal patterns of individual diseases along with its respective frequency and severity” [37].

While global warming has proven harmful to some pathogens, climate change is largely increasing the risk of infectious diseases. Added to this is the possibility that pathogens may become increasingly resistant to heat, becoming extremely dangerous for humans as they will be able to survive one of our body’s main defense mechanisms: fever.

On the basis of these observations, it is necessary to interrupt – as far as is within man’s possibilities – this chain of disastrous events for the planet and for human health. The awareness that the survival of humanity is inseparably connected to that of the planet must push humanity and governments to adopt radically different lifestyles and sustainable consumption models to slow down and keep under control those climate changes that can lead to an increase in the spread of pathogens and their resistance to our natural defenses and drugs.
